# 肺癌介入专题前言

**DOI:** 10.3779/j.issn.1009-3419.2020.101.08

**Published:** 2020-06-20

**Authors:** 兰军 张

**Affiliations:** 1 State Key Laboratory of Oncology in South China, Collaborative Innovation Center for Cancer Medicine, Guangzhou 510000, China; 2 510000 广州，中山大学附属肿瘤医院胸外科 Department of Thoracic Surgery, Sun Yat-sen University Cancer Center, Guangzhou 510000, China

肺癌是全球发病率和致死率均居首位的癌症^[1]^，而肺癌介入已经成为肺癌诊断和治疗中不可或缺的重要组成部分。肺癌介入主要分为经气道介入、经胸廓介入和经血管介入。经气道介入主要包括以支气管镜为基础的技术，经胸廓介入则主要包括经胸廓穿刺定位、射频消融、微波消融和冷冻消融等微创介入的手段，而经血管介入则主要用于晚期肺癌大量咯血和上腔静脉综合征的姑息治疗。

作为肺癌经气道介入的主要手段，支气管镜除了被广泛用于肺癌手术切除前的常规气道评估，还能进行气道细胞学刷检和组织学活检以明确病理学诊断。另外，超声支气管镜还能用于纵隔淋巴结的评估和穿刺活检，能大大提高肺癌临床分期的准确性，有助于让肺癌患者接受更为合适的治疗。近年来，随着低剂量螺旋计算机断层扫描（computed tomography, CT）的普及，越来越多的肺部外周结节在体检筛查中被发现，然而绝大部分发现的肺结节是良性的^[2]^。因此，这些肺结节的良恶性鉴别与治疗前准确定位尤为重要。近年飞速发展的电磁导航支气管镜就很好地满足了这一需求，电磁导航技术使支气管镜不再局限于段支气管以上的气道，并让支气管镜准确到达外周肺结节。同时，支气管镜还能标记肿瘤位置，有利于放疗的设计与手术切除的定位^[3]^。对于晚期肺癌，支气管镜下治疗还能作为一种姑息治疗的有效手段，支气管镜能实现直接对肿瘤注射化疗药物、植入放射性粒子、支气管镜下消融、光动力治疗、放置气管支架等局部治疗，从而达到直接减瘤、杀灭肿瘤细胞、减少全身毒性和姑息治疗的效果^[4]^。总的来说，近年技术的发展让支气管镜准确到达病灶的能力大大升高，随之提高的还有其对肺癌诊断的准确性和治疗的精准性。虽然目前大部分支气管镜相关的最新技术依然处于探索阶段，它们的灵敏度、特异性、疗效和安全性依然有待进一步验证，但希望不久的将来这些技术能成为肺癌诊断和治疗的有力武器。

肺癌经胸廓介入手段主要包括诊断和治疗两大方面，在诊断方面，CT引导下肺穿刺已经成为获取肺癌病理诊断的重要手段，这是大部分无法接受手术切除的肺癌患者获取病理诊断的有效途径^[5]^。而在治疗方面，消融治疗，包括射频消融、微波消融和冷冻消融可以作为无法接受手术切除的肺癌的局部控制手段，目前已经有大量动物和临床试验^[6]^证实了肺癌消融治疗的可行性，但其安全性和有效性仍待进一步验证，希望其能成为补充手术和放疗的肺癌局部控制有力治疗手段。

肺癌经血管介入主要用于晚期肺癌的姑息治疗，其具体应用包括对肺癌大量咯血和上腔静脉综合征的治疗。肿瘤的异常血管增生可能会导致患者咯血，引起患者大量咯血的动脉大部分为支气管动脉，因而采用支气管动脉栓塞能有效控制患者大量咯血的症状^[7]^。另一方面，上腔静脉综合征主要是由于肿瘤的外部压迫或者是血管内癌栓形成，因此置入上腔静脉支架能快速缓解患者头面部肿胀等主观不适，提升生活质量^[8]^。

借《中国肺癌杂志》肺癌介入专题的机会，希望更多同行了解肺癌介入的现状和发展前景，更希望广大研究者积极投身到肺癌介入的研究，为肺癌介入的发展添砖加瓦。
